# Supplementary material for: Nitrogen fertilizer application for improving the biomass, quality, and nitrogen fixation of alfalfa (Medicago sativa L.) at different growth stages in a saline‒alkali soil
Source: PeerJ. 2025 Jan 16;13:e18796. doi: 10.7717/peerj.18796 (PMC11742254; doi:10.7717/peerj.18796)
Supplement: Supplemental Information 1 [file peerj-13-18796-s001.docx]

**Supplementary materials**

**Table. S1 Results of ANOVA for parameters among N fertilizer application rates and dates （2020）**

| **Parameters** | **N fertilizer application rate** | | | | | **Date** | | | | | **N fertilizer application rate × Date** | | | |
| --- | --- | --- | --- | --- | --- | --- | --- | --- | --- | --- | --- | --- | --- | --- |
|  | **df** | | ***F*** | ***P-value*** | | **df** | | ***F*** | ***P-value*** | | | **df** | ***F*** | ***P-value*** |
| height | 4 | | 0.789 | 0.541 | | 2 | | 116.951 | *p<0.001* | | | 8 | 0.228 | 0.983 |
| Stem diameter | 4 | | 0.916 | 0.467 | | 2 | | 114.611 | *p<0.001* | | | 8 | 0.778 | 0.625 |
| Branching number | 4 | | 0.253 | 0.906 | | 2 | | 66.079 | *p<0.001* | | | 8 | 0.989 | 0.464 |
| Biomass | 4 | | 2.266 | *p<0.05* | | 2 | | 56.562 | *p<0.001* | | | 8 | 0.581 | 0.785 |
| Stem to leaf | 4 | | 3.481 | *p<0.05* | | 2 | | 72.881 | *p<0.001* | | | 8 | 1.674 | 0.146 |
| Leaf N concentration | 4 | | 5.202 | *p<0.01* | | 2 | | 10.332 | *p<0.001* | | | 8 | 1.615 | 0.162 |
| Stem N concentration | 4 | | 2.132 | *p<0.05* | | 2 | | 1.814 | 0.1804 | | | 8 | 2.379 | 0.101 |
| Root N concentration | 4 | | 4.839 | *p<0.01* | | 2 | | 0.291 | 0.749 | | | 8 | 1.068 | 0.411 |
| Shoot N uptake | 4 | | 2.709 | *p<0.05* | | 2 | | 33.334 | *p<0.001* | | | 8 | 0.467 | 0.8691 |
| Crude Protein | 4 | | 6.479 | *p<0.001* | | 2 | | 16.148 | *p<0.001* | | | 8 | 2.821 | *p<0.05* |
| Acid Detergent Fiber | 4 | | 2.795 | *p<0.05* | | 2 | | 36.076 | *p<0.001* | | | 8 | 1.808 | 0.114 |
| Neutral Detergent Fiber | 4 | | 0.527 | 0.716 | | 2 | | 15.031 | 0.103 | | | 8 | 1.997 | 0.0815 |
| Soil NH_4_^+^-N concentration (0-20cm) | 4 | 1.282 | | | 0.2991 | | 2 | 14.005 | | *p<0.001* | | 8 | 2.796 | *p<0.05* |
| Soil NH_4_^+^-N concentration (20-40cm) | 4 | 4.164 | | | *p<0.01* | | 2 | 5.783 | | *p<0.01* | | 8 | 1.770 | 0.122 |
| Soil NO_3_^-^-N concentration (0-20cm) | 4 | 6.429 | | | *p<0.001* | | 2 | 7.499 | | *p<0.01* | | 8 | 1.491 | 0.202 |
| Soil NO_3_^-^-N concentration (20-40cm) | 4 | 7.084 | | | *p<0.001* | | 2 | 3.010 | | 0.064 | | 8 | 0.549 | 0.810 |

**Table. S2 Results of ANOVA for parameters among N fertilizer application rates and dates（2021）**

| **Parameters** | **N fertilizer application rate** | | | | | **Date** | | | | | **N fertilizer application rate × Date** | | | |
| --- | --- | --- | --- | --- | --- | --- | --- | --- | --- | --- | --- | --- | --- | --- |
|  | **df** | | ***F*** | ***P-value*** | | **df** | | ***F*** | ***P-value*** | | | **df** | ***F*** | ***P-value*** |
| height | 4 | | 2.055 | 0.096 | | 6 | | 480.901 | *p<0.001* | | | 24 | 0.887 | 0.617 |
| Stem diameter | 4 | | 5.023 | *p<0.01* | | 6 | | 92.487 | *p<0.001* | | | 24 | 1.405 | 0.137 |
| Branching number | 4 | | 6.752 | *p<0.001* | | 6 | | 316.465 | *p<0.001* | | | 24 | 1.277 | 0.213 |
| Biomass | 4 | | 8.191 | *p<0.001* | | 6 | | 78.488 | *p<0.001* | | | 24 | 0.753 | 0.779 |
| Stem to leaf | 4 | | 0.874 | *p<0.05* | | 6 | | 86.187 | *p<0.001* | | | 24 | 1.486 | 0.102 |
| Leaf N concentration | 4 | | 5.426 | *p<0.001* | | 6 | | 56.316 | *p<0.001* | | | 24 | 1.714 | 0.076 |
| Stem N concentration | 4 | | 0.924 | 0.455 | | 6 | | 155.321 | *p<0.001* | | | 24 | 1.281 | 0.211 |
| Root N concentration | 4 | | 4.266 | *p<0.01* | | 6 | | 5.871 | *p<0.001* | | | 24 | 1.185 | 0.2859 |
| Shoot N uptake | 4 | | 7.786 | *p<0.001* | | 6 | | 18.568 | *p<0.001* | | | 24 | 0.616 | 0.908 |
| Crude Protein | 4 | | 2.393 | *p<0.001* | | 6 | | 181.856 | *p<0.001* | | | 24 | 1.788 | *p<0.05* |
| Acid Detergent Fiber | 4 | | 1.390 | *p<0.001* | | 6 | | 35.599 | *p<0.001* | | | 24 | 1.509 | 0.094 |
| Neutral Detergent Fiber | 4 | | 3.030 | *p<0.05* | | 6 | | 41.113 | *p<0.001* | | | 24 | 1.272 | 0.216 |
| n-fixed-tpos | 4 | | 2.868 | *p<0.05* | | 6 | | 6.355 | *p<0.001* | | | 24 | 0.704 | 0.686 |
| %Ndfa | 4 | | 2.482 | *p<0.05* | | 6 | | 14.246 | *p<0.001* | | | 24 | 0.495 | 0.850 |
| nodule number | 4 | 1.595 | | | *p<0.05* | | 6 | 14.142 | | *p<0.001* | | 24 | 1.765 | 0.065 |
| Soil NH_4_^+^-N concentration (0-20cm) | 4 | 1.546 | | | 0.198 | | 6 | 23.061 | | *p<0.001* | | 24 | 2.047 | *p<0.05* |
| Soil NH_4_^+^-N concentration (20-40cm) | 4 | 1.805 | | | 0.137 | | 6 | 26.090 | | *p<0.001* | | 24 | 3.117 | *p<0.001* |
| Soil NO_3_^-^-N concentration (0-20cm) | 4 | 9.398 | | | *p<0.001* | | 6 | 7.905 | | *p<0.001* | | 24 | 1.901 | *p<0.05* |
| Soil NO_3_^-^-N concentration (20-40cm) | 4 | 5.481 | | | *p<0.001* | | 6 | 8.783 | | *p<0.001* | | 24 | 1.136 | 0.331 |
