# Supplementary material for: Nitrogen fertilizer application for improving the biomass, quality, and nitrogen fixation of alfalfa (Medicago sativa L.) at different growth stages in a saline‒alkali soil
Source: PeerJ. 2025 Jan 16;13:e18796. doi: 10.7717/peerj.18796 (PMC11742254; doi:10.7717/peerj.18796)
Supplement: Supplemental Information 11 [file peerj-13-18796-s011.docx]

**Codebook: Translations of Non-English Terms in Figure_2.R and Figure_3.R**

| **Non English language** | **English translations** |
| --- | --- |
| 字符 | Character |
| 加显著性字母并调整位置 | Add significance letters and reposition |
| 调整X轴顺序 | Adjustment of X-axis order |
| 分面 | Facet |
